# Psychometric Properties of the Persian Version of Dizziness Handicap Inventory

**DOI:** 10.22038/ijorl.2019.38094.2252

**Published:** 2019-11

**Authors:** Robabeh Soleimani, Mir Mohammad Jalali, Babak Bakhshayesh, Pasha Rashidi Mojdehi, Seyed Mohammad Sadegh Ghadiri Asli

**Affiliations:** 1 Kavosh Cognitive Behavioral Sciences and Addiction Research Center, School of Medicine, Guilan University of Medical Sciences, Rasht, Iran.; 2 Otorhinolaryngology Research Center, School of Medicine, Guilan University of Medical Sciences, Rasht, Iran.; 3 Department of Neurology, Poursina Hospital, Guilan University of Medical Sciences, Rasht, Iran.; 4 Faculty of Medicine, Guilan University of Medical Sciences, Rasht, Iran.

**Keywords:** Dizziness, Dizziness Handicap Inventory, Questionnaire, Reliability, Validity

## Abstract

**Introduction:**

The present study was designed to investigate the psychometric properties of the Persian version of the Dizziness Handicap Inventory (P-DHI). In addition, this research was targeted toward assessing the association of P-DHI with Medical Outcome Study 36-Item Short Form Health Survey (SF-36) and Hospital Anxiety and Depression Scale (HADS). The current study also involved a comparison of the scores of patients and healthy participants and implementation of a factor analysis.

**Materials and Methods:**

This cross-sectional study was conducted on 113 patients with dizziness and 30 healthy individuals referring to tertiary centers for otolaryngology and neurology, affiliated to Guilan University of Medical Sciences, Rasht, Iran. The mean age of the patients was 44.5±13.6 years. All patients re-completed the P-DHI after 2 weeks. Internal consistency and reproducibility of the inventory were evaluated using the Cronbach’s alpha coefficient, Bland-Altman limits of agreement, and intraclass correlation coefficients. In addition, the relationships of the P-DHI with SF-36 and HADS were evaluated using the Spearman correlation coefficient. An exploratory factor analysis was also run to determine the factor structure of the questionnaire.

**Results:**

The Cronbach’s alpha coefficient of P-DHI scale was obtained as 0.86. In addition, the functional, physical, and emotional subscales of this instrument had the Cronbach’s alpha coefficients of 0.76, 0.52, and 0.80, respectively. The limits of agreement were 16 points for the total scale, and the range of intraclass correlation coefficients was 0.90-0.96. The P-DHI showed a fair correlation with vertigo severity which assesses functional disability subscale. This scale also demonstrated a moderate correlation with SF-36 and HADS. Factor analysis revealed a 2-factor solution which was different from the factor structure of the original DHI.

**Conclusion:**

As the findings indicated, the P-DHI had good psychometric properties; therefore, it could serve as a useful tool for measuring disability in patients with dizziness and unsteadiness.

## Introduction

Vertigo is a frequent and highly uncomfortable complaint. Vestibular disorders account for about one-fourth of cases suffering from this condition. Vertigo can be severe enough to affect patients’ occupational performance or daily activities (1). Hansson proposed vestibular rehabilitation as a valuable treatment for these patients (2). Researchers used several outcome measures (e.g., vestibular-ocular reflex, balance performance, and performance on computerized dynamic posturography or functional ability) to evaluate the handicapping effects of dizziness on the quality of life. However, none of these tests reflect the real impact of dizziness on patients’ activities. 

Jacobson and Newman developed the Dizziness Handicap Inventory (DHI) to assess the impact of vestibular vertigo/dizziness on the quality of life and measure the results of vestibular rehabilitation therapy (3). So far, the DHI has been translated into different languages, and its psychometric properties have been evaluated in different populations. In Iran, this questionnaire was translated by Jafarzadeh et al. (4), performing a study on patients with dizziness aged 18-70 years. It was shown that the Persian version of DHI (P-DHI) had good face and content validities. The total scale, along with its subscales, had the Cronbach's alpha coefficient range of 0.79-0.90. 

The primary aim of the present study was to evaluate the validity and reliability of the P-DHI and examine its dimensions using an exploratory factor analysis. 

To assess the disability and feelings of anxiety and depression, we evaluated the association of DHI, original subscale, and retained factors in our factor analysis with functional disability and several generic questionnaires. The secondary aim was to compare the P-DHI scores between subjects with and without vertigo.

## Materials and Methods

**Study population**

Patients who had dizziness for at least one month were recruited from the Otorhinolaryngology Clinic of Amiralmomenin Hospital and Neurology Clinic of Poursina Hospital, affiliated to Guilan University of Medical Sciences, Rasht, Iran, between May 2016 and August 2018. The inclusion criteria were: 1) age of 18-75 years, 2) ability to walk, 3) ability to perform at least 50% of daily activities, and 4) fluency in speaking Persian. On the other hand, the patients with dizziness due to cardiopulmonary, musculoskeletal, or psychiatric disorders were excluded from the study. 

Healthy participants were selected from family members and health care staff. In line with research ethics principle, informed consent was obtained from all included subjects. In addition, the study protocol was approved by the Ethical Committee of Guilan University of Medical Sciences. All procedures were also performed in accordance with the principles of the Helsinki Declaration. A total of 113 patients (i.e., 48 males and 65 females; mean age: 44.5±11.3 years) met the inclusion criteria and participated in the investigation at the first and second examinations (after 2 weeks). 

In addition, 30 healthy subjects (i.e., 16 males and 14 females; mean age: 43.8±9.9 years) underwent only the first examination. Out of the included patients, 50, 37, and 26 subjects had a peripheral vestibular disorder, a central vestibular pathology, and signs/symptoms of both sites classified as a mixed syndrome, respectively. In addition to comprehensive history taking, a neuro-otological examination and laboratory tests were performed. Laboratory tests consisted of audiometry, tympanometry, acoustic reflex, and video nystagmography.

**Procedure**

**Dizziness Handicap Inventory**

The original DHIis a 25-item questionnaire evaluating patient's physical, functional, and emotional limitations. Each item has three responses of yes (4 points), sometimes (2 points), or no (0 point). The range of DHI score is from 0 (no disability) to 100 (severe disability) (3). The Persian version of the DHI was prepared using the back-translation method. Two independent medical experts with very good knowledge in English translated the original version in Persian, and then reached a consensus on the final translated version. Then, two independent translators that were unfamiliar with the original DHI retranslated the Persian version of DHI. Ten patients with complaints of dizziness filled out the pre-final Persian version of DHI questionnaire. They comprehended all the items and found no difficulty in understanding and answering the questions.

**Perception of dizziness or instability**

The patients were asked to display their sensation of dizziness or instability at the time of evaluation on a visual analog scale (VAS). This scale consisted of a straight line, measuring 100 mm, where 0 represented the absence of symptoms and 100 signified their highest perception of symptoms.

**Modified Berg Balance Scale**

The Modified Berg Balance Scale (mBBS) was used to assess balance condition in the participants. Two items, related to static sitting and standing balance, were omitted from the original tool. The mBBS comprises 12 balance-related tasks. Each task is given a score range of 0 (unable) to 4 (independent) (5).Clinicians apply the mBBS to evaluate the different stages of rehabilitation in patients with acute or chronic balance problems. The mBBS has been established as a valid tool to estimate balance condition in younger or older patients of both genders.

**Iranian Medical Outcome Study 36-Item Short Form Health Survey**

The interval level scoring for all eight scales ranges from 0 (for worse health status) to 100 (for the best possible health status as measured by the questionnaire). The Iranian version of the SF-36 comprises 36 items covering eight health-related concepts, including physical functioning, role physical, body pain, general health, vitality, social function, role emotional, and mental health. Each scale is scored individually within a score range of 0 (for poor health) to 100 (for better health) (6). The total score is estimated by averaging the scores of eight scales.

**Hospital Anxiety and Depression Scale**

This 14-item questionnaire comprises two subscales which assess the non-somatic symptoms of anxiety (HADS-A) and depression (HADS-D). Each item of the questionnaire is rated from 0 to 3. The two subscales had a score range of 0 (no sign of anxiety or depression) to 21 (maximum level of anxiety or depression). A score of 11 or greater is indicative of probable anxiety or depression (7). 

**Statistical analysis**

A sample size with a minimum of 100 subjects is required for studies investigating the psychometric properties of questionnaires by means of factor analysis. In addition, and a sample size of 50 is considered adequate for determining test-retest reliability (8). We assessed the frequency of floor and ceiling effects; in this regard, the questionnaires were considered standard when the frequency of floor or ceiling effects was less than 15% (9).The validation of a questionnaire requires checking the metric characteristics, including internal consistency, test-retest reliability, and construct validity (10).

The Bland and Altman method was used to assess absolute agreement between the first and second administration of the P-DHI (11). For high repeatability, 95% of the difference scores should fall within ±2 standard deviations of the zero difference score. Moreover, the associations of the P-DHI factors with the SF-36, HADS, VAS, and other physical examination results were estimated. Spearman’s correlations were interpreted according to the Domholdt. In this respect, 0.00-0.25, 0.26-0.49, 0.50-0.69, 0.70-0.89, and 0.90-1.00 were suggestive of very weak, weak, moderate, strong, and very strong correlations, respectively (12).

Furthermore, the construct validity of the P-DHI was examined using explanatory factor analyses with principal component analysis (PCA) and varimax rotation. The suitability of the sample was evaluated by means of the Bartlett test of sphericity. 

The value for this test was less than 0.001 (χ^2^=1565, df=300), indicating that correlations in the dataset are appropriate for factor analysis. In addition, sampling adequacy was assessed using the Kaiser-Meyer-Olkin (KMO) measurement. 

The KMO ranges from 0 to 1.0, and the overall KMO must be 0.60 or higher to perform factor analysis. In this study, the KMO value was estimated at 0.77. The number of factors to be extracted is an important decision in a factor analysis. In this study, we used a parallel analysis and the eigenvalue one test (>1) or Kaiser criterion. Since factor analysis is an exploratory technique, it is recommended to consider these techniques as suggestions. Analyses should be run with more and fewer factors (13). We retained items if they had loadings greater than 0.40. No more than 50% of the residuals should be greater than 0.05 (14). 

Since items of the DHI are categorical, categorical PCA (CATPCA) was performed in order to confirm the dimensional structure. Original variables that had the component loadings of ≥0.70 across the selected principal components were selected. The internal consistency of the retained factors was investigated by estimating Cronbach’s alpha coefficients and corrected item-total correlations (CI-TCs). All analyses were performed using the SPSS (version 21.0) and STATA (version 13.0).

## Results

**Study population**

A total of 113 patients with a mean age of 44.5±13.6 years participated in this study (Table 1). None of the participants obtained a score of less than 16 or more than 72 in the questionnaire. The mean total score was 33.2, and in the majority of the participants (74.3%), the impact of dizziness was considered moderate. The mean scores for physical, functional, and emotional subscales were 15.1 (0-28), 13.9(0-36), and 4.2 (0-36),respectively. 

As indicated, the score was higher for the physical domain than for the emotional and functional domains. A comparative study was conducted on the scores of P-DHI and its subscales in the dizzy patients and 30 healthy controls. The results of Mann-Whitney U tests showed significantly higher scores in the patient group (P<0.001). This finding indicated that the P-DHI had strong reliability in the patients with dizziness.

**Internal consistency**

The Cronbach’salpha coefficient of the total scale (r=0.86) showed that the P-DHI have excellent internal consistency in the Iranian population. The coefficients of the functional, physical, and emotional subscales were estimated at 0.76, 0.52, and 0.80, respectively. There was a strong positive correlation between the total P-DHI score and its subscales (r>0.75). The values of the CI-TC coefficients ranged from 0.24 to 0.75 with the lowest and highest values belonging to items P11 and E9/E23, respectively. All CI-TC values were higher than the recommended value of 0.20 (15).

**Test-retest reliability**

We administrated the questionnaire again averagely 13.1 days later. The results of repeatability coefficients for the total scale and its subscales showed that 96.0%, 97.0%, 93.1%, and 90.1% of the differences for the functional, physical, and emotional subscales were between 2 standard deviations, respectively.

**Fig 1:**
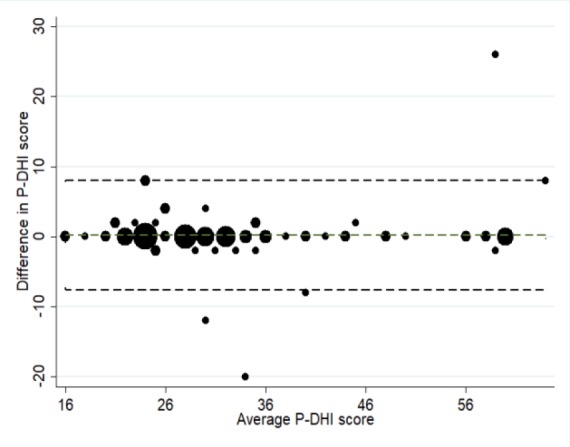
Bland–Altman plot which compared differences between the P-DHI scores at test and retest. Pitman's test of difference in variance was insignificant (P = 0.21)

 The analysis of the test-retest reliability was accomplished using the interclass correlation coefficient (ICC). The results showed a high degree of ICC in the total score and all subscale scores of the Persian version of DHI (all ICCs>0.90). The plot of the difference of paired variables versus their average of the P-DHI scores in the first and second tests (Bland-Altman plot) showed no measurement error (Fig.1). 

**Table 1:** Baseline characteristics of the study population

Characteristics of the participants	Patients (n=113)	Healthy (n=30)
**Age (mean±SD) (y)**	44.5**±**11.3	43.8**±**9.9
**Gender n (%)**		
** Male** ** Female**	48 (42.5) 65 (57.5)	16 (53.3) 14 (46.7)
**Speech Reception Threshold (mean±SD) (dB)**	26.0 (13.9)	22.0 (9.7)
**Groups of diagnosis n (%)**		
** Peripheral** ** Central** ** Mixed**	50 (44.3) 37 (32.7) 26 (23.0)	
**Duration of dizziness (mean±SD)**	8.0**±**8.5	
**Level of disability n (%)** ** Mild (<40)** ** Moderate (40-70)** ** Severe (>70**	23 (20.4) 84 (74.3) 6 (5.3)	
**The Persian version of DHI**		
** Total (mean±SD)** ** Physical subscale (mean±SD)** ** Functional subscale (mean±SD)** ** Emotional subscale (mean±SD)**	33.2**±**12.8 15.1**±**3.9 13.9**±**6.2 4.2**±**5.2	15.8**±**3.6 8.3**±**3.7 4.9**±**2.8 2.6**±**1.3
**HADS**		
** Total (mean±SD)** ** Anxiety subscale (mean±SD)** ** Depression subscale (mean±SD)**	9.5**±**3.4 3.5**±**3.0 5.9**±**3.8	1.8**±**1.5 0.5**±**0.6 1.3**±**2.3
**mBBS (mean±SD)**	6.5**±**5.3	1.9**±**2.4
**SF 36 (mean±SD)**	70.3**±**11.3	87.7**±**7.0

**DHI: Dizziness Handicap Inventory, HADS: Hospital Anxiety and Depression Scale, mBBS: Modified Berg Balance Scale, SF-36:** **SF-36: Medical Outcome Study 36-Item Short Form Health Survey**

**Construct validity**

To estimate the construct validity of the P-DHI, we examined Pearson correlation between total P-DHI score and its subscales with several measures. The mean VAS for vertigo severity was obtained as 45.9±15.5. In 42 cases (41.6%), VAS was higher than 50. There was a weak correlation (r=0.44; P<0.01) between the P-DHI and the vertigo severity. The Kruskal-Wallis ANOVA with the three levels of vertigo severity showed the significant relationship of self-reported disability and the P-DHI total score (P=0.02), as well as the functional and emotional subscales (P=0.004 and P=0.006, respectively). The association of P-DHI with SF-36 and HADS was clearly higher than 0.60 and moderate. The mean score of the SF-36 was estimated at 70.3 (range: 30.3-87.8). Pearson correlations of the total and subscale scores of the P-DHI with the eight scales of the SF-36 were consistent. Among the P-DHI subscales, the functional scale showed a better association with all the SF-36 subscales. The anxiety and depression subscales of the HADS had a score range of 0-26 (mean=9.5). The results showed probable anxiety and depression in 3 (2.7%) and 15 cases (13.3%), respectively.

The results of the Mann-Whitney U test revealed a significantly higher score of P-DHI in the participants with probable anxiety or depression (P=0.03 and P<0.01, respectively). As shown in Table 2, there was a direct and moderate correlation between total P-DHI and the mBBS score, as well as the P-DHI subscales and the mBBS score (r=0.66 and *r* range=0.42-0.67, respectively).

**Internal validity-factor analysis**

**Fig2:**
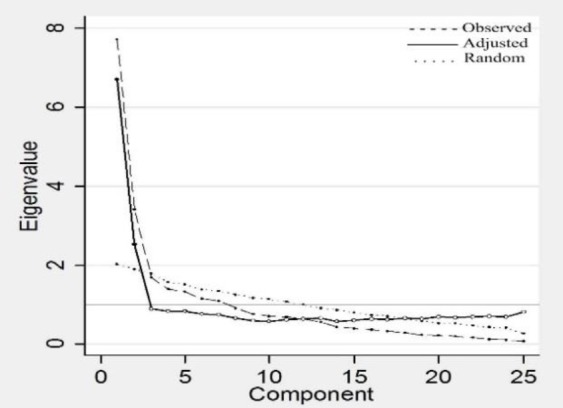
The results of the parallel analysis of a PCA. Non-retained components are marked with a hollow circle on the adjusted eigenvalues curve

An exploratory factor analysis was run to evaluate the internal validity of the questionnaire. The Guttman-Kaiser Criterion showed a 6-factor solution, which explained 74.0% of the variance. Because 4 loaded factors had less than 3 items, further investigation was not considered. The parallel analysis using Horn’s criterion indicated that two components should be retained (Fig. 2). Furthermore, the screeplot indicated 2- and 3-factor solutions.

**Table 2:** Results of the principal component analysis of the 25 items of the Persian version of the Dizziness Handicap Inventory

Subscale	Item	Three-factor solution			Two-factor solution	
		1	2	3	1	2
**P1**	Looking up	0.51			0.53	
**E2**	Feeling frustrated			0.50	0.51	
**F3**	Restricted business or recreational travel	0.71			0.71*	
**P4**	Walking down a supermarket aisle	0.68			0.70	
**F5**	Getting into or out of bed		0.91			0.91*
**F6**	Restricted participation in social activities		0.57			0.59
**F7**	Difficulties in reading	0.54			0.54	
**P8**	Ambitious activities, sports, and dancing		0.90			0.90*
**E9**	Afraid of leaving home alone	0.78			0.78*	
**E10**	Embarrassed in front of others			0.62	0.36	
**P11**	Quick head movements		0.89			0.90
**F12**	Avoid heights		0.68			0.69
**P13**	Turning over in bed		0.93			0.93
**F14**	Strenuous housework or yard work		0.75			0.77*
**E15**	Afraid people think you are intoxicated			0.26	-0.14	
**F16**	Go for a walk by yourself	0.82			0.80*	
**P17**	Walking down a sidewalk	0.87			0.87*	
**E18**	Difficult to concentrate	0.80			0.79*	
**F19**	Walking around the house in the dark	0.61			0.56	
**E20**	Afraid to stay at home alone	0.74			0.71*	
**E21**	Feeling handicapped	0.69			0.67	
**E22**	Stressed relationships with family/friends	0.55			0.56	
**E23**	Depressed	0.82			0.82*	
**F24**	Job or household activities	0.53			0.50	
**P25 **	Bending over		0.79			0.80

**Asterisk indicates component loadings with values of ≥ 0.70 in CATPCA.**

**Three-factor solution**

The 3-factor solution accounted for 60.5% of the variance. The eigenvalues for the first to third factors were 9.8, 3.9, and 1.4, respectively. The evaluation of the fit of the model showed 115 (38.0%) non-redundant residuals. The range of the communality values was about 0.7. A total of 14 items were loaded on the first factor. The factor loadings of 10 out of 14 were greater than 0.6 (Table.3). Six and five items belonged to the original emotional and functional subscales, respectively. It seemed that the first factor reflects “the effect of dizziness and unsteadiness on emotion”. The moderate associations of this factor with vertigo severity (r=0.52), the mBBS score (r=0.57), and especially the HADS-Depression subscale (r=0.71) confirmed our proposed description. The second factor contained 8 items, all of which had a factor loading of> 0.6 (Table.3). This factor revealed the physical and functional disability. The third item was loaded on the third factor. However, factor loadings greater than 0.6 were observed only in one item. The Cronbach’s alpha value of the third factor was obtained as 0.17 which was less than the commonly accepted minimal standards of 0.7. There was only a fair association between the third factor and the items assessing the quality of life (Table.2). The weak to fair correlation could be explained by a limited number of items in the third factor. However, there liability of this factor was doubtful.

**Table 3:** Association of the Persian version of the Dizziness Handicap Inventory with vertigo characteristics and other questionnaires

	***Duration of vertigo (mo)***	***Vertigo severity***	***Berg score***	***SF-36 score***	***HADS score (anxiety)***	***HADS score (depression)***
*P-DHI total*	0.42**	0.44**	0.71**	-0.79**	0.64**	0.77**
*Physical subscale*	0.35**	0.22*	0.63**	-0.72**	0.57**	0.66**
*Functional subscale*	0.22*	0.38**	0.72**	-0.75**	0.57**	0.72**
*Emotional subscale*	0.44**	0.47**	0.49**	-0.60**	0.63**	0.62**
*Factor 2*						
* Component 1*	0.32**	0.54**	0.58**	-0.66**	0.64**	0.72**
* Component 2*	0.31**	0.08	0.71**	-0.76**	0.52**	0.64**
*Factor 3*						
* Component 1*	0.29**	0.52**	0.57**	-0.63**	0.63**	0.71**
* Component 2*	0.25**	0.11	0.72**	-0.76**	0.49**	0.63**
* Component 3*	0.46**	0.18	0.20**	-0.33**	0.47**	0.33**

Values are Spearman correlation coefficients: ** correlation is significant at 0.01 level (1-tailed); * correlation is significant at 0.05 level (1-tailed). Boldface indicates moderate associations.

P-DHI: Persian version of the Dizziness Handicap Inventory, HADS: Hospital Anxiety and Depression Subscale, SF-36: Medical Outcome Study 36-Item Short Form Health Survey

**Two-factor solution**

The two-factor solution accounted for 54.8% of the variance. The fit of the model evaluation showed 137 (45.0%) non-redundant residuals. Items of the first factor were similar to factors 1 and 3 of the previous solution. The mean values of communalities were about 0.5. In addition, the items of factor 2 were identical to the items of factor 2 in the previous solution. The factor loadings of 9 items of the first factor and 7 items of the second factor were greater than 0.6. Similar to the 3-factor solution, moderate associations existed between the first factor and vertigo severity (r=0.54), SF-36, and HADS subscales (Table.2). The second factor showed moderate to strong associations with the SF-36 (r=-0.76) and the anxiety and depression subscales of the HADS (r=0.52 and r=0.64, respectively). 

There was a significant correlation between the P-DHI total score and SF-36. Additionally, moderate correlations (range: 0.27-0.59) existed between the eight health-related concepts of the SF-36 and the first factor of the P-DHI. This finding supports that factors 1 and 2 mainly focus on the emotional and physical effects of dizziness on a patient's general health, respectively. Our results revealed that the 2-factor solution was the most reliable solution which had clinically relevant dimensions. The Cronbach’s alpha coefficients of the first and second factors were 0.91 and 0.91, respectively. Moreover, all CI-TC values within eachfactor were higher than 0.2.Since the items of the DHI are categorical, the CATPCA was performed. The loadings of the first two components of the CATPCA model explained 47.1% of variance. The first and second factors loaded with 17 and 8 items, respectively. All the component loadings, except for E15, were positive in the first factor. The five items in the first factor had loadings of > 0.7. Three out of eight items in the second factor had a high positive loading (Table.3). In agreement with PCA, the results of CATPCA confirmed the stability of the factors. 

## Discussion

In the current study, the original DHI was translated into the Persian language and the validity, reliability, and factorial structure of the P-DHI were evaluated. In terms of the DHI total scale scores, a mean value of 33.2±12.8 was found for the entire distribution of the scores, suggesting that the patients generally reported low to moderate effects of vertigo on their daily activities. Similar to the original English version, Cronbach’s alpha values of the P-DHI total scale and its functional and emotional subscales reached 0.7, which is the commonly accepted minimal standard (3). 

In contrast with previous studies, the results of factor analysis demonstrated good internal consistency for two components in the 2-factor solution. This discrepancy can be due to differences in the sample and cultural factors. All CI-TCs obtained for the items in the 2-factor solution were higher than the recommended minimum value of 0.2. This finding is indicative of the accuracy of P-DHI just like that of the original test. The relative test-retest reliability of the total score of the P-DHI, and its subscales was excellent as indicated by the ICC values of> 0.90. This reliability is in accordance with that of the original version of DHI. The Bland-Altman plot did not show a systematic pattern; in other words, differences seemed to be random. 

In this study, about 44% and 33% of the patients had peripheral or central vestibular disorders, respectively. This ratio has been reported differently across various studies. The study populations investigated by Kurre et al. (16) and Poon et al. (17) seem to be more comparable with our study population. The P-DHI total scale showed 16 points for the limits of agreement. In contrast with our result, Jacobson and Newman (3) indicated 18 points for the English version of the DHI. In this study, we assessed the correlation of the P-DHI with generic questionnaires (e.g., SF-36 and HADS). There were moderate associations which supported the good convergent validity of the P-DHI. In addition, the association between P-DHI and mBBS was moderate. This finding is in agreement with the results of previous studies (18-20). 

It is a merit to indicate low to fair relationship between the P-DHI and self-perceived disability (vertigo severity). In 1980, the World Health Organization published the International Classification of Impairments, Disabilities and Handicaps (ICIDH). This organization revised the ICIDH in 1999 and defined handicap as the level of participation (21). "Participation is the nature and extent of a person's involvement in life situations in relationship to impairments, activities and contextual factors." We believe the DHI measure various subscales such as emotions which can be very handicapping. However, VAS for vertigo severity addresses the level of activity (disability).Our results showed the internal validity of the 2-factor solution, in which the first factor mainly consisted of emotional and functional items, and the second factor predominantly included physical items. Similar to our findings, A smundson et al. (22) and Vereeck et al. (23) reported two components. However, other researchers showed more than three components in the internal validity of DHI (4,16,17). The discrepancy between the results of our study and those of previous research may be due to the selected method for making a decision regarding item retention. It is known that the Kaiser’s “eigenvalue greater than 1” rule sometimes overestimates the actual number of factors to retain. Horn’s parallel analysis (PA) is an adaptation of the Kaiser criterion, which uses information from random samples, and a strong consensus exists on PA as the most accurate method (24). Despite the importance of factor retention decisions, only Vereeck et al. (23) utilized parallel analysis.

Our findings support the results of a study carried out by Jafarzadeh (4).Furthermore, we applied a confirmatory factor analysis to find functional relationships between the measures of different constructs. Reliability refers to the consistency or repeatability of a set of measurements. Reliability is tested by the test-retest method and measures of internal consistency. To measure internal consistency, we used the intraclass correlations in addition to the Cronbach’s alpha coefficient. The most important strength of our study is the application of parallel and factor analyses to assess the DHI. This facilitated the evaluation of the latent construct and measurement of reliability and validity among components. Moreover, we repeated factor analysis using categorical PCA that supported the stability of a dimension assessed by PCA. 

One of the limitations of this study was that its sample size was small for performing sub-analyses between patients with different diagnosis (peripheral versus central) and within different levels of vertigo disability. Therefore, the results should be interpreted with caution. 

## Conclusion

Our results revealed that the P-DHI have good reliability and validity in the Iranian population. This study confirmed the measurement properties of the P-DHI as a discriminative and evaluative tool. Therefore, the P-DHI could be used to evaluate the course of disease and response to treatment in Iranian patients. The exploratory factor analysis revealed a 2-factor solution. Our proposed model should be studied in the future in greater detail by means of structural equation modeling.
